# Limited prognostic value of revised tumour deposit definition in tumour node metastasis (TNM)8 in colorectal cancer: national cohort study

**DOI:** 10.1093/bjsopen/zraf148

**Published:** 2026-01-21

**Authors:** Frida Stoltz, Simon Lundström, Pamela Buchwald

**Affiliations:** Department of Surgery, Skåne University Hospital, Malmö, Sweden; Department of Clinical Sciences, Lund University, Lund, Sweden; Department of Surgery, Skåne University Hospital, Malmö, Sweden; Department of Clinical Sciences, Lund University, Lund, Sweden; Department of Surgery, Skåne University Hospital, Malmö, Sweden; Department of Clinical Sciences, Lund University, Lund, Sweden

**Keywords:** colorectal neoplasms, neoplastic deposit, tumour staging, prognosis

## Abstract

**Background:**

Tumour deposits are an important prognostic factor in colorectal cancer. In tumour node metastasis (TNM)8, the definition became stricter as TNM7’s previous requirement for absence of lymphatic tissue was expanded to also include nerve and vascular tissue. TNM8 has been criticised for its limited prognostic value. This study aimed to compare prognostic differences for patients with colorectal cancer with tumour deposits staged with TNM7 and TNM8.

**Methods:**

This national retrospective cohort study included patients with colorectal cancer who underwent surgical resection in 2011–2014 and 2017–2019. Exclusion criteria were metastatic stage IV disease, non-radical or non-curative surgery, unstated tumour deposit status, or early (≤ 30 days) mortality. Univariable, multivariable, and interaction term Cox regression analyses examined differences in overall survival and distant metastasis between TNM7 and TNM8 stagings. Multivariable models were adjusted for age, gender, American Society of Anesthesiologists score, number of positive lymph nodes, TNM stage, neoadjuvant, and adjuvant treatment.

**Results:**

Of 19 413 patients operated on during 2011–2014 and 15 027 during 2017–2019, 23 966 were included. The TNM7 cohort had 1225 (9.5%) patients with tumour deposits, and the TNM8 cohort had 1407 (12.7%). There was an improved 5-year distant metastasis-free survival for patients with tumour deposits (hazard ratio 2.35 (95% confidence interval 2.14 to 2.58)) in the TNM8 cohort, but no benefit in overall survival, compared with patients in the TNM7 cohort. Interaction analysis revealed no prognostic difference associated with tumour deposit status between the two TNM editions.

**Conclusion:**

Despite increased complexity, the revised definition of tumour deposits in TNM8 did not enhance prognostic ability compared with TNM7.

## Teaser

This national retrospective cohort study highlights that TNM8’s new definition of tumour deposits has added increased complexity and reduced reproducibility without enhancing prognostic ability compared with TNM7. The definition requires further refinements in future TNM editions.

## Introduction

Colorectal cancer (CRC) is the third most common malignancy worldwide and the second leading cause of cancer-related mortality^1^. CRC staging follows the tumour node metastasis (TNM) system, a widely used classification that provides critical prognostic information^2^. This system is continuously updated by the American Joint Committee on Cancer (AJCC) and the Union for International Cancer Control (UICC) to incorporate emerging clinical and pathological evidence^3^.

Tumour deposits (TDs) have gained increasing recognition as an important prognostic factor in CRC^4^. A 2016 international meta-analysis^4–6^ reported that TDs were present in 4.9–41.8% of CRC cases, and they were strongly associated with poorer survival and higher risk of distant metastasis (DM) The increased risks have been proven to be independent of lymph node status^7^. Since the initial inclusion in TNM5 (1997), the definition of TDs has evolved with each TNM edition^8^. In TNM7 (2009)^9^, TDs were defined as extra-nodal tumour tissue in pericolic or perirectal adipose tissue without histological evidence of residual lymph nodes. Simultaneously, TDs were incorporated into the N category as N1c, representing lymph node-negative, TD-positive CRC^9^. In TNM8 (2016)^10^, TDs were further refined by excluding nodules containing vascular and neural structures, aiming to improve classification accuracy. However, concerns have been raised about the evidence underlying the current definition, as well as the risk for low inter- and intraobserver agreement among pathologists^8^. As a result, the prognostic benefit of the revision can be questioned.

This study aimed to compare the prognostic value of TDs on overall and DM survival among patients with CRC staged according to TNM7 *versus* TNM8. The hypothesis was that the refinements introduced in TNM8 would not result in clinically significant differences in prognostic outcomes compared with TNM7.

## Methods

### Study design

This study was based on data from the Swedish Colorectal Cancer Register (SCRCR), a national registry founded in Sweden in 1995 to systematically collect data on patients with CRC. SCRCR is one of the most comprehensive cancer registries and covers 98.5% of patients with CRC in Sweden^11^. The registry contains extensive details about patient demographic, tumour characteristics, TNM stage, administrated treatment, and follow-up data of diagnosed patients. Follow-up data are collected continuously and in a standardised manner 3 and 5 years after surgery. This study was approved by the Swedish Ethical Review Authority (DNR 2023-02433-02 & DNR 2023-07143-01) and conducted in accordance with national and international guidelines for research ethics. All included patients provided consent for participation in the SCRCR. Inclusion in the Swedish Cause of Death Register is mandatory by Swedish law.

### Study population

The study included Swedish patients diagnosed with colorectal adenocarcinoma who underwent acute or elective surgical resection for CRC. Patients meeting any of the following criteria were excluded; synchronous or metachronous CRC, non-curative surgery, metastatic stage IV disease, non-radical (R1 or R2) surgery, unstated TD status, or early (≤ 30 days) mortality. R1 was defined as a surgical resection specimen, including lymph nodes and TDs, with tumour tissue present in the microscopic margins (<0.1 mm) according to the pathologist. After exclusion, patients were stratified based on the TNM edition corresponding to the year of diagnosis; TNM7 for patients diagnosed between 2011 and 2014 and TNM8 for patients diagnosed between 2017 and 2019. Due to the lack of exact transition dates for each individual centre, the years 2015 and 2016 were excluded to ensure clear separation and minimize inconsistencies during the transition period between the classification systems. Within each TNM edition, patients were further categorised by TD status (TD-negative or TD-positive).

### Data collection

Data were extracted from the SCRCR for two different time periods: 1 January 2011 to 31 December 2014 and 1 January 2017 to 31 December 2019. Mortality and emigration status was obtained from the Swedish Cause of Death Register. Data extraction was performed on 2 September 2020 for the 2011–2014 group, and on 18 January 2025 for the 2017–2019 group.

### Outcomes of interest

The primary outcome was predictive differences of TDs on overall survival (OS) and DM survival between TNM7 and TNM8. OS was defined as death from any cause, as registered in the Swedish Cause of Death Register. DM was defined as cancer recurrence located outside the peritoneal cavity, at intraperitoneal sites distant from the primary tumour, or within non-regional lymph nodes. Follow-up time was defined as the time from surgery to last known vital status.

### Statistics

Categorical variables were presented as numbers and percentages (%), whereas continuous variables were reported as medians with interquartile ranges (25–75th percentile). Differences in clinical characteristics were evaluated by comparing TNM7 and TNM8 separately within the TD-negative and TD-positive groups. Pearson's chi-square test were used for categorical variables and the Mann-Whitney U test for continuous and ordinal variables. Kaplan-Meier curves were used to compare OS and DM over 5 years for patients who were TD-negative and TD-positive, based on TNM version. The log rank test was used to evaluate statistical differences between the groups.

The association between TD status and oncological outcomes was analysed using both uni- and multivariable regression analyses. Multivariable regression analyses were adjusted for possible confounding variables that were identified using directed acyclic graphs (*Fig. S1*). Variables identified as possible confounding factors were age, sex, American Society of Anesthesiologists (ASA) score, number of positive lymph nodes, TNM stage, neoadjuvant treatment, and adjuvant treatment. Results were reported as a hazard ratio (HR) with a 95% confidence interval (c.i.). The prognostic impact of TNM edition on TD status was further investigated using interaction term Cox regression analysis within the multivariable regression analysis. An additional sensitivity analysis was performed with adjustment for tumour location (colon/rectum).

Patients with missing data due to emigration were censored at the time of emigration for all outcomes in both Kaplan–Meier and Cox regression analyses. Deceased patients were censored for DM at the day of death. Only patients with complete information were included in the multivariable Cox regression analysis.

All statistical analyses were performed using R version 4.4.1 (R Core Team, 2024 with RStudio Team, 2024, Vienna, Austria). Kaplan–Meier curves and Cox regression analyses were created using the *survival* package^12^ and visualizations were generated using the *ggplot2* package^13^. *P* ≤ 0.05 was considered to indicate statistical significance.

The study was approved by the Swedish Ethical Review authority (DNR: DNR: 2020–01769 and DNR: 2023-02433-02).

## Results

### Study population

**Fig. 1:**
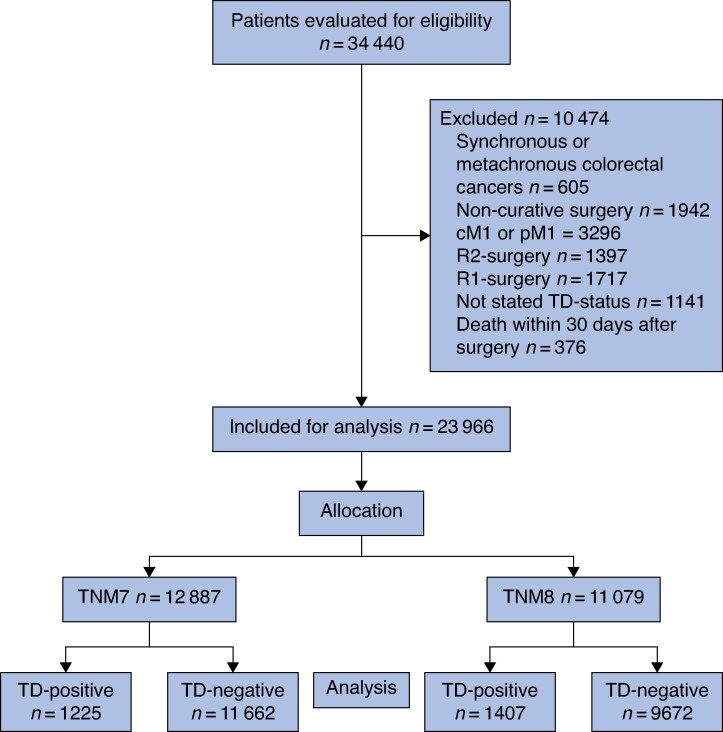
Study flow chart illustrating patient selection process for inclusion in study cM1 and pM1, clinical and pathological M1-staged; R2-surgery, macroscopical residual tumour after surgery; R1-surgery, microscopical residual tumour after surgery; TD, tumour deposit; TNM7/8, tumour node metastasis edition 7/8.

A total of 34 440 patients met the inclusion criteria and were assessed for eligibility. After applying the exclusion criteria, 23 966 patients remained for analysis. Of these, 12 887 were diagnosed according to the TNM7 system, with 1225 (9.5%) classified as TD-positive, and 11 079 were diagnosed according to the TNM8 system, with 1 407 (12.7%) classified as TD-positive (*Fig. 1*).

Patient characteristics, stratified by TNM version and TD status, are presented in *Table 1*. Sex, age, and BMI were similar between the two TNM cohorts. The TNM8 cohort had a higher proportion of patients with colon cancer, a higher average ASA score, and was more frequently discussed at preoperative multidisciplinary conferences compared with the TNM7 cohort. Patients who were TD-positive generally had higher clinical and pathological TNM stages and were more likely to have received neoadjuvant and adjuvant therapy than patients who were TD-negative. Patients who were both TD-positive and TD-negative assessed according to TNM8 received neoadjuvant and adjuvant therapy less frequently than patients assessed according to TNM7.

**Table 1:** Patient clinical characteristics stratified by TNM version and TD status

	TD-negative			TD-positive		
	TNM7 (*n* = 11 662)	TNM8 (*n* = 9672)	P*	TNM7 (*n* = 1225)	TNM8 (*n* = 1407)	P*
**Sex**						
Male	6125 (52.5%)	5035 (52.1%)	0.509	653 (53.3%)	768 (54.6%)	0.537
Age (years), median (i.q.r.)	72 (65–79)	73 (66–80)	< 0.001	71 (63–79)	72 (64–79)	0.360
BMI (kg/m^2^), median (i.q.r.)	25 (23–28)	26 (23–29)	< 0.001	25 (23–28)	26 (23–29)	0.508
Missing	808 (6.9%)	290 (3.0%)		69 (5.6%)	33 (2.3%)	
**Tumour localization**			< 0.001			0.027
Colon	7808 (67.0%)	6981 (72.2%)	<0.001	801 (65.4%)	978 (69.5%)	0.400
Right	4422 (56.6%)	4292 (61.5%)		400 (49.9%)	508 (51.9%)	
Left	3381 (43.3%)	2675 (38.3%)		401 (50.1%)	470 (48.1%)	
Rectal	3854 (33.0%)	2691 (27.8%)	0.050	424 (34.6%)	429 (30.5%)	0.701
Low (0–5 cm)	1112 (28.9%)	711 (26.4%)		90 (21.2%)	99 (23.1%)	
Mid (6–10 cm)	1560 (40.5%)	1127 (41.9%)		190 (44.8%)	187 (43.6%)	
High (11–15 cm)	1133 (29.4%)	822 (30.5%)		138 (32.5%)	139 (32.4%)	
**ASA score**			< 0.001			0.011
ASA 1–2	7981 (68.4%)	5980 (61.8%)		862 (70.4%)	919 (65.3%)	
ASA 3	3116 (26.7%)	3091 (32.0%)		313 (25.6%)	415 (29.5%)	
**cT category**			< 0.001			< 0.001
T1–T2	2586 (22.2%)	3150 (32.6%)		138 (11.3%)	237 (16.8%)	
T3	4223 (36.2%)	3875 (40.1%)		571 (46.6%)	708 (50.3%)	
T4	1109 (9.5%)	1215 (12.6%)		177 (14.4%)	287 (20.4%)	
TX	3571 (30.6%)	1432 (14.8%)		326 (26.6%)	175 (12.4%)	
**cN category**			< 0.001			0.081
N0	6169 (52.9%)	5396 (55.8%)		408 (33.3%)	457 (32.5%)	
N1-2	3455 (29.6%)	3634 (37.6%)		596 (48.7%)	829 (58.9%)	
NX	1774 (15.2%)	642 (6.6%)		192 (15.7%)	121 (8.6%)	
**cStage**			0.002			0.605
Stage I	2049 (17.6%)	2539 (26.3%)		89 (7.3%)	152 (10.8%)	
Stage II	2253 (19.3%)	2169 (22.4%)		195 (15.9%)	257 (18.3%)	
Stage III	3452 (29.6%)	3657 (37.8%)		596 (48.7%)	837 (59.5%)	
Missing	3908 (33.5%)	1299 (13.4%)		345 (28.2%)	160 (11.4%)	
**Neoadjuvant therapy**			< 0.001			< 0.001
Yes	2919 (25.0%)	1637 (16.9%)		413 (33.7%)	339 (24.1%)	
**Preoperative MDC**			< 0.001			< 0.001
Yes	10 156 (87.1%)	9342 (96.6%)		1134 (92.6%)	1354 (96.2%)	
**pT category**			0.003			0.013
T0	152 (1.3%)	24 (0.2%)		7 (0.6%)	1 (0.1%)	
T1	1228 (10.5%)	951 (9.8%)		16 (1.3%)	14 (1.0%)	
T2	2469 (21.2%)	2251 (23.3%)		77 (6.3%)	79 (5.6%)	
T3	6529 (56.0%)	5146 (53.2%)		769 (62.8%)	848 (60.3%)	
T4	1258 (10.8%)	1298 (13.4%)		355 (29.0%)	465 (33.0%)	
**pN category**			0.183			0.015
N0	8486 (72.8%)	7029 (72.7%)		0 (0%)	0 (0%)	
N1a	1079 (9.3%)	1094 (11.3%)		174 (14.2%)	221 (15.7%)	
N1b	970 (8.3%)	861 (8.9%)		262 (21.4%)	313 (22.2%)	
N1c	0 (0%)	0 (0%)		312 (25.5%)	392 (27.9%)	
N2a	568 (4.9%)	435 (4.5%)		214 (17.5%)	240 (17.1%)	
N2b	400 (3.4%)	207 (2.1%)		254 (20.7%)	231 (16.4%)	
**pStage**			0.768			0.346
Stage I	2949 (25.3%)	2688 (27.8%)		0 (0%)	0 (0%)	
Stage II	5107 (43.8%)	4284 (44.3%)		0 (0%)	0 (0%)	
Stage III	3009 (25.8%)	2586 (26.7%)		1212 (98.9%)	1360 (96.7%)	
**Positive lymph nodes**			< 0.935			0.010
Lymph node count, median (i.q.r)	0 (0, 1)	0 (0, 1)		2 (0, 6)	2 (0, 5)	
**Examined lymph nodes**			< 0.001			0.013
< 12	1366 (11.7%)	687 (7.1%)		103 (8.4%)	83 (5.9%)	
**CRM (mm)**			0.681			0.280
> 1.0	9549 (81.9%)	9312 (96.3%)		1029 (84.0%)	1266 (90.0%)	
0.1–1.0	363 (3.1%)	343 (3.5%)		95 (7.8%)	136 (9.7%)	
Missing	1750 (15.0%)	17 (0.2%)		101 (8.2%)	5 (0.4%)	
**Adjuvant therapy**			< 0.001			< 0.001
Yes	2881 (24.7%)	1972 (20.4%)		799 (65.2%)	770 (54.7%)	
**Distant metastasis**			< 0.001			0.015
Yes	1449 (12.4%)	1005 (10.4%)		455 (37.1%)	458 (32.6%)	
**Deceased**			< 0.001			< 0.001
Yes	4002 (34.3%)	2729 (28.2%)		634 (51.8%)	645 (45.8%)	

All data presented as *n* (%) unless stated otherwise. Missing data of less than 5% were excluded from the table for clarity but were accounted for in the analysis. TNM, tumour node metastasis; TD, tumour deposit; i.q.r., interquartile range; BMI, body mass index; ASA, American Society of Anesthesiologists; MDC, multidisciplinary conference; CRM, circumferential resection margin. *P-values are presented for differences between patients who were TD-positive classified according to TNM7 and TNM8, as well as for differences between patients who were TD-negative classified according to TNM7 and TNM8.

The overall mortality rate among patients who were TD-positive was 51.8% in the TNM7 cohort and 45.8% in the TNM8 cohort. For patients who were TD-negative, the overall mortality rate was 34.3% in the TNM7 cohort and 28.2% in the TNM8 cohort. DM occurred in 37.1% of patients who were TD-positive in the TNM7 cohort and 32.6% in the TNM8 cohort. Among patients who were TD-negative, DM occurred in 12.4% in the TNM7 cohort and in 10.4% in the TNM8 cohort.

### Survival analyses

**Fig. 2:**
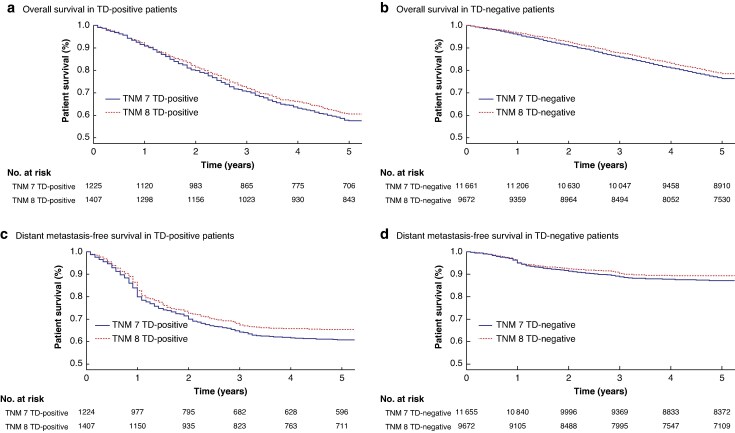
Kaplan–Meier analyses of 5-year overall survival and distance metastasis-free survival for patients with CRC based on TD status and TNM version used for diagnosis Five-year overall survival is presented for patients classified as **a** TD-positive (*P* = 0.14) and **b** TD-negative (*P* = 0.00012). Distant metastasis-free survival is presented for patients classified as **c** TD-positive (*P* = 0.023) and **d** TD-negative (*P* < 0.0001). Patients that emigrated were censored at the date of emigration. Statistical significance was assessed using the log rank test, with *P* < 0.05 defined as significant. TNM, tumour node metastasis; TD, tumour deposit.

The 5-year OS and DM-free survival for patients with TDs stratified by the TNM edition is presented in *Fig. 2*. Patients who were TD-positive diagnosed with TNM8 showed no differences in OS over a 5-year period but demonstrated an improved 5-year DM-free survival (48.7% *versus* 50.5%; *P* = 0.023) compared with those diagnosed with TNM7. Comparably, TD-negative patients showed both better OS (76.4% *versus* 77.9%; *P* = 0.044) and DM-free survival (71.8% *versus* 73.5%; *P* < 0.001) when diagnosed with TNM8 compared with those diagnosed with TNM7.

The results from the Cox regression analyses are presented in *Table 2*. Both univariable and multivariable regression analyses revealed that patients who were TD-positive had an increased risk of poorer OS (multivariable HR 1.88 (95% c.i. 1.74 to 2.05)) and DM (multivariable HR 2.35 (95% c.i. 2.14 to 2.58)) compared with patients who were TD-negative. For the interaction analysis, TNM8 alone had a protective effect on OS (HR 0.83 (95% c.i. 0.78 to 0.89)) and DM (HR 0.88 (95% c.i. 0.80 to 0.97)). However, when examining the combined effect of TNM8 and TD-positive status, no significant differences were observed between the two cohorts for OS or DM.

**Table 2:** Univariable, multivariable, and interaction term regression analysis of overall survival and distant metastasis for patients with CRC stratified by TD status

	Overall survival HR	Distant metastasis HR
**Univariable analysis**		
TD^−^	1	1
TD^+^	2.08 (1.95, 2.22)	3.68 (3.41, 3.98)
**Multivariable analysis**		
TD^−^	1	1
TD^+^	1.88 (1.74, 2.05)	2.35 (2.14, 2.58)
**Interaction term analysis**		
TD^+^	1.77 (1.57, 1.99)	2.28 (2.00, 2.59)
TNM8	0.83 (0.78, 0.89)	0.88 (0.80, 0.97)
TNM8, TD^+^	1.14 (0.98, 1.34)	1.08 (0.91, 1.29)

Values in parentheses are 95% confidence intervals. Multivariable analyses and interaction term analyses are adjusted for the confounding factors: age, sex, ASA-score, number of positive lymph nodes, neoadjuvant treatment and adjuvant treatment. HR, hazard ratio; CRC, colorectal cancer; TD, tumour deposit; TNM, tumour node metastasis.

Interaction term sensitivity analysis, with inclusion of tumour location (*Table S1*), continued to show poorer OS (HR 1.77 95% c.i. (1.57 to 2.00)) and increased risk of DM (HR 2.27 (95% c.i. 2.00 to 2.59)) in patients who were TD-positive compared with patients who were TD-negative. TNM8 maintained its protective effect on OS (HR 0.83 (95% c.i. 0.77 to 0.89)) and DM (HR 0.88 (95% c.i. 0.80 to 0.97)), whereas the interaction term between TD status and TNM edition remained non-significant for both OS and DM.

## Discussion

This retrospective study compared the prognostic value of TDs in patients with CRC staged according to TNM7 and TNM8. Whereas TNM8 was associated with a modestly increased protective effect on OS and DM, it was not explained by the revised TD classification. Instead, the improved outcomes observed in patients who were TD-positive under TNM8 are more likely attributed to broader improvements, such as advancements in surgical techniques and enhanced oncological treatment.

TNM8 introduced a more stringent definition of TDs by excluding nodules containing vascular or neural structures, aiming to improve the staging system’s prognostic accuracy^10^. However, this revision has faced criticism on several fronts. First, the increased complexity of the new classification has resulted in reduced interobserver agreement^14^. Another author^14^ emphasized this issue, noting significant variability in TD identification, which raises concerns about the reproducibility and prognostic reliability of TNM8’s TD criteria. Second, the biological rationale behind the revision has been questioned, as the origin and metastatic pathway of TDs remain uncertain^15^. Studies^4^ suggests that the presence of vascular and neural structures in TDs contributes to the poorer prognosis. Solid scientific evidence, which is currently lacking, is warranted to justify the exclusion of such nodules.

An additional concern related to the TNM8 definition is the risk of stage migration^16^. Under TNM8, non-lymphatic tumour nodules with vascular or neural structures are downgraded from pN1c (stage III) to pN0 (stage II), as they are no longer considered as TDs^8^. This reclassification should theoretically decrease the number of N1c-cancers in favour of N0. However, in the present study, the proportion of patients who were TD-positive was higher in the TNM8 cohort than in the TNM7 cohort, and the staging between pN0 and pN1c was similar across both cohorts. These findings suggests that the expected stage migration did not occur. The lack of shift in staging distribution may reflect increased awareness of TDs, advancements in pathology training, or other developments that inevitably have occurred during the study period, such as the watch-and-wait regime, rather than an actual shift^17,18^. These changes may have counteracted the effects on the stricter TD definition in TNM8. Interestingly, despite similar N category distribution, patients who were TD-positive that were staged according to TNM8 received adjuvant therapy less frequently. Given that previous studies^19,20^ have shown significant benefits of adjuvant treatment in patients with colon cancer who were TD-positive, the reduced use of adjuvant therapy may have masked potential improvements and may explain the lack of observed OS benefit in the TNM8 cohort. Adjuvant therapy in rectal cancer has declined internationally, partly due to the introduction of total neoadjuvant treatment and the limited evidence of benefit in this subgroup^21^. In Sweden, gradual implementation of screening programmes has led to earlier detection, further reducing the need for adjuvant therapy^22^.

To assess whether the revised definition of TDs in TNM8 contributed to the observed differences in outcomes, an interaction term analysis between TD status and TNM edition was performed. Whereas TNM8 independently demonstrated a protective effect on OS and DM, the interaction term between TD status and TNM edition did not show any significant difference in prognostic impact of TDs between TNM7 and TNM8. Thus, the improved outcomes observed in the TNM8 cohort are likely not attributable to the revised TD definition alone. Instead, other factors not accounted for in the analysis, such as advances in surgical techniques and enhanced oncological treatment, are more likely to have contributed to these improvements^19,23^. As the stratification of the study population was time-dependent, year of surgery could not be included in the sensitivity analysis as this would constitute double adjustment for time.

The present study population included both colon and rectal cancer, which often are considered two distinct cancer forms as they differ in both biology and treatment approach^24,25^. However, extending the interaction term analysis to include tumour location did not alter the results, suggesting that the non-significant interaction term remains robust across tumour locations.

The proportion of patients excluded due to R1 and R2 resections differed between cohorts. R2-based exclusions were lower in the TNM8 cohort, whereas R1-based exclusions were higher. These differences may indicate underlying variations in surgical techniques or preoperative assessment between the original cohorts. As these patients were excluded from the study population, no further adjustment for these variables were performed.

This study includes several limitations. The underlying differences in surgical techniques and in preoperative assessment between the patient groups could imply selection bias. Ideally, prognostic differences would be assessed by applying both TNM definitions to the same population; however, the retrospective design and large cohort size made this resource-intensive approach unfeasible. Furthermore, the increasing awareness of the prognostic significance of TDs over time may have influenced the likelihood of their detection, introducing a potential detection bias between cohorts. Another limitation of this study is that TD classification is based on registry data, which were assessed by a single pathologist at the time of surgery. As more recent studies have demonstrated a high intraobserver variability in the histological assessment of TDs, a re-evaluation of the histological specimen could have increased the robustness of the results.

Overall, this study found that the increased complexity of the revised TD definition in TNM8 did not enhance prognostic ability for patients who were TD-positive compared with TNM7. Given the increased complexity, lower reproducibility, and lack of clear prognostic advantage for patients who were TD-positive under TNM8, the definition of TDs warrants thorough revision and further refinement in the forthcoming TNM9 edition.

## Supplementary Material

zraf148_Supplementary_Data

## Data Availability

All data used are available through the Swedish Colorectal Cancer Registry.

## Context and relevance

This study compared the prognostic value of tumour deposits in patients with CRC staged according to TNM7 and TNM8. The updated definition introduced in TNM8 has been criticized for lower interobserver agreement and questionable exclusion of certain nodules as tumour deposits. Whereas TNM8 was independently associated with a protective effect in OS and DM, the interaction analysis revealed no prognostic difference associated with tumour deposit status between the two TNM editions, and future editions should take in consideration these findings.

## Twitter summary

TNM8’s definition of tumour deposits brings increased complexity and lower reproducibility, without offering better prognostic value. The definition should be revised before introducing TNM9.
