# Supplementary material for: Limited prognostic value of revised tumour deposit definition in tumour node metastasis (TNM)8 in colorectal cancer: national cohort study
Source: BJS Open. 2026 Jan 21;10(1):zraf148. doi: 10.1093/bjsopen/zraf148 (PMC12822601; doi:10.1093/bjsopen/zraf148)
Supplement: zraf148_Supplementary_Data [file zraf148_supplementary_data.zip › Supplementary_Materials.docx]

**The limited prognostic value of the revised tumour deposit definition in TNM8 for colorectal cancer: a national cohort study**

Frida Stoltz (F.S.) Research student**^1,2^**, Simon Lundström (S.L.) MD, PhD student**^1,2^**, Pamela Buchwald (P.B) MD, Ass.Prof**^1,2^**.

**Affiliations**

1. Department of Surgery, Skåne University Hospital, Malmö, Sweden
2. Department of Clinical Sciences, Lund University, Lund, Sweden

**Correspondence**

Frida Stoltz, Research student.
Address: Department of Surgery, Skåne University Hospital, SE-214 21, Malmö, Sweden.

Email: [soc14fla@student.lu.se](mailto:soc14fla@student.lu.se)
Telephone: +4670-8805633

ORCID ID: 0009-0002-0930-816X

**Supplementary Materials - Index**

| **Supplementary Tables and Figures** |  |
| --- | --- |
| Supplementary Table 1 | *page 2* |
| Supplementary Figure 1 | *page 3* |
|  |  |

**Supplementary Table 1.** Hazard ratio of the sensitivity analysis of the effect of tumour location on overall survival and distant metastasis.

|  | **Overall Survival** | **Distant Metastasis** |
| --- | --- | --- |
| **Interaction-term analysis** | HR | HR |
| **TD^+^** | 1.77 (1.57-2.00) | 2.27 (2.00-2.59) |
| **TNM8** | 0.83 (0.77-0.89) | 0.88 (0.80-0.97) |
| **TNM8 + TD^+^** | 1.15 (0.98-1.34) | 1.08 (0.91-1.28) |

Sensitivity analysis of tumour location on CRC patients’ overall survival and distant metastasis in interaction-term analyses of TD status and TNM edition. Adjusted for the confounding factors; age, sex, ASA-score, tumour location, number of positive lymph nodes, neoadjuvant treatment and adjuvant treatment. TD, tumour deposits; HR, hazard ratio.

**
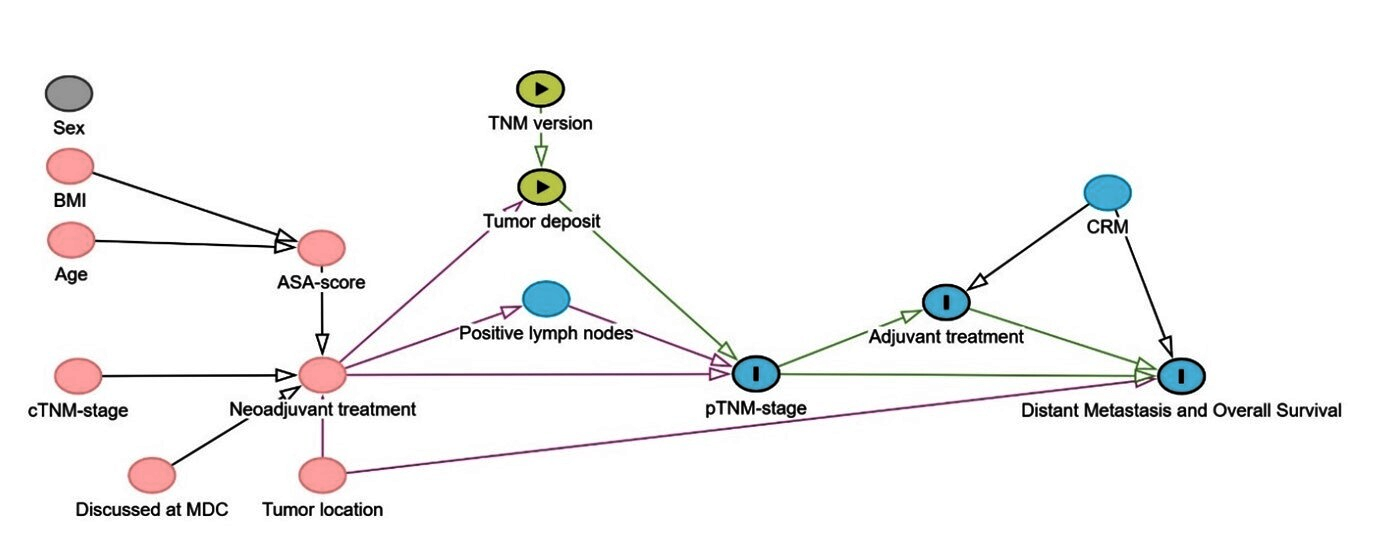
**

**Supplementary Figure 1.** **Direct Acyclic Graph***.* Illustration of relationships between exposures, outcomes and covariates. Pink nodes represent ancestors of exposure and outcome, green nodes represent exposure, while blue nodes represent outcomes. Black arrows represent direct effects, green arrows represent causal paths, while pink arrows represent biasing paths. TNM, Tumour-Node-Metastasis; cTNM, clinical TNM; pTNM, pathological TNM; CRM, Circumferential resection margin, MDC; Multidisciplinary conference.
